# Genome mosaic structure of two novel HIV-1 recombinant forms (CRF01_AE/B) in men who have sex with men in Hebei, China

**DOI:** 10.1186/s12981-023-00527-x

**Published:** 2023-05-25

**Authors:** Xinli Lu, Wenbin Gao, Yingying Wang, Meng Liu, Ning An, Yan Li, Yuqi Zhang, Qi Li

**Affiliations:** 1 Department of AIDS Research, Hebei Key Laboratory of Pathogen and Epidemiology of Infectious Disease, Hebei Provincial Center for Disease Control and Prevention, Shijiazhuang, Hebei China; 2 College of Life Science, Cangzhou Normal University, Cangzhou, Hebei China

**Keywords:** HIV-1, Subtype, Unique recombinant form, MSM

## Abstract

**Background:**

Homosexual contact is the main route of human immunodeficiency virus type one (HIV-1) transmission in Cangzhou Prefecture, Hebei, China. Moreover, the number of circulating recombinant forms (CRFs) and unique recombinant forms (URFs) in this key population is ever increasing.

**Methods:**

In this study, we identified two novel URFs (hcz0017 and hcz0045) from two men who have sex with men (MSM) based in Cangzhou Prefecture. Phylogenetic and recombinant breakpoint analyses, based on the near full-length genomes (NFLGs) of the two novel URFs, showed that they originated from a recombination between HIV-1 CRF01_AE and subtype B.

**Results:**

HXB2 numbering revealed that the NFLGs of hcz0017 and hcz0045 each contained the following seven subregions: hcz0017: I_B_ (790–1,171 nt), II_CRF01_AE_ (1,172–2,022 nt), III_B_ (2,023–4,469 nt), IV_CRF01_AE_ (4,470–5,866 nt), V_B_ (5,867–7,462 nt), VI_CRF01_AE_ (7,463–8,379 nt), VII_B_ (8,380–9,411 nt); hcz0045: I_CRF01_AE_ (790–5,147 nt), II_B_ (5,148–5,614 nt), III_CRF01_AE_ (5,615–6,035 nt), IV_B_ (6,036–6,241 nt), V_CRF01_AE_ (6,242–7,325nt), VI_B_ (7,326–8,254 nt), VII_CRF01_AE_ (8,255–9,411 nt). Moreover, the two MSM from whom the novel URFs originated from were diagnosed as recently HIV-1-infected, suggesting that the high prevalence of HIV-1 among MSM was related to high-risk sexual activity such as unprotected anal sex and multiple sexual partners.

**Conclusions:**

Our results highlight the need to continually monitor HIV-1 diversity in Hebei and its neighboring provinces to achieve a more effective control of HIV-1 spread within the MSM community.

## Background

Four decades have elapsed since the discovery of the first case of acquired immune deficient syndrome (AIDS) [1] in 1981 in the USA. Since then, the causal agent of AIDS, the human immunodeficiency virus (HIV), and HIV type 1 (HIV-1) in particular, has spread worldwide, causing a serious global public health problem. According to an official report [2] released by the Joint United Nations Program on HIV/AIDS, 37.7 million people were living with HIV worldwide in 2020, despite the success of HIV control efforts. HIV-1 genetic diversity is complex and constantly evolving. To date, 118 HIV-1 circulating recombinant forms (CRFs) have been registered in the HIV Sequence Database (https://www.hiv.lanl.gov/components/sequence/HIV/search/search.html) and numerous unique recombinant forms (URFs) have also been identified [3]. A global survey of 383,519 samples from 116 countries found that the three most common HIV-1 subtypes globally were C, B, and A, accounting for 69% of all HIV isolates [4]. However, recombinant HIV-1 strains are more frequently observed in China than in other countries, with CRF07_BC and CRF01_AE as the dominant strains [5]. HIV-1 diversity can impact the results of diagnostic assays, viral load determination, and the development of HIV vaccines [4, 6]. Therefore, the continued surveillance of HIV-1 subtypes is essential.

Hebei Province in North China, covers 190,000 km^2^ and comprises 11 prefectures. At the end of October 2020, a total of 17,891 individuals with HIV-1/AIDS were reported in the whole province [7]. Among the newly reported cases, the proportion of homosexual transmission had increased from 41.8% to 2010 to 64.8% in 2020 in Hebei. Moreover, because of this shift in HIV-1 transmission routes (from blood to sexual contact), the most prevalent HIV-1 subtype in Hebei switched from subtype B (before 2005) to CRF01_AE (after 2005) [8, 9]. However, subtype B has remained in circulation in Hebei and now constitutes the third most prevalent subtype in this province [9]. Thus, the cocirculation and dual infection (via the sexual route of transmission) with the CRF01_AE and subtype B strains will undoubtedly provide opportunities for recombination between strains.

Of the 11 prefectures in Hebei, Cangzhou, which shares a border with Tianjin, is severely affected by HIV-1. Since the first case of HIV-1 infection in 2003, more than 100 HIV-1-positive cases across the eight counties of Cangzhou have been reported. Homosexual contact plays a key role in HIV-1 spread in Cangzhou Prefecture [10], with 77.6% of HIV-1 infections observed in individuals aged 15–49 years [10]. However, few studies of URFs have been conducted in the HIV-1-infected population of Cangzhou Prefecture.

In the present study, two novel URFs (hcz0017 and hcz0045), which originated from a recombination between CRF01_AE and subtype B, were detected in two recently infected men who have sex with men (MSM) based in Cangzhou Prefecture, Hebei, China by near full-length genome (NFLG) sequence analysis.

## Materials and methods

### Study participants

The hcz0017 URF originated from a divorced 55-year-old man, whereas hcz0045 was derived from an unmarried 23-year-old man (Table 1). Both subjects were infected with HIV-1 through homosexual contact, and their CD4^+^ T cell counts and viral loads were over 300 cells/mm^3^ and 3 × 10^4^ copies/ml, respectively. The individuals were confirmed as anti-HIV-1-antibody-positive by western blotting in November 2019 and were diagnosed as having recently acquired HIV-1 infection using a limiting antigen avidity enzyme immunoassay, CD4^+^ T cell counts, and viral load quantification. Blood samples were obtained from the two donors during their voluntary counseling and testing appointments. Written informed consent was obtained from both HIV-1 individuals prior to blood collection. The study was approved by the Local Ethics Committee of Hebei Provincial Center for Disease Control and Prevention (No. IRB(S)2020-031).

**Table 1:** Baseline information of two individuals recently infected with HIV-1

Sample ID	Gender	Age	Prefecture	Marital status	Infection route	First CD4(cells/mm^3^)	Sample source(copies/ml)	Viral load	Final OD-n ^a^
hcz0017	Male	55	Cangzhou	Divorced	MSM	303	VCT	30,800	0.53
hcz0045	Male	23	Cangzhou	Unmarried	MSM	333	VCT	2,240,000	0.79

a, OD value of two individuals in HIV-1 detection of the recent infections. VCT, voluntary counseling and testing

### Quantification of CD4 + T cell counts and HIV-1 viral load

CD4^+^ T cell counts were determined using the FACSCount System (Becton–Dickinson, Franklin Lakes, NJ, USA) from 50 µL of whole blood. HIV-1 viral load was quantified using the Amplicor HIV-1 Monitor test (COBAS TaqMan 48; Roche, Switzerland).

### HIV-1 genome characterization

An HIV-1 NFLG assay was performed as previously described [11]. Raw sequences were assembled using Contig Express 9.1. Multiple sequence alignment with Clustal W and manual editing were performed using Bio-Edit 7.0 software. The standard reference sequences of HIV-1 subtypes were downloaded from the HIV Databases (http://www.hiv.lanl.gov/content/index), including all full-length CRF sequences associated with 01/B recombination. The neighbor-joining (N-J) phylogenetic trees were constructed using the Kimura two-parameter model with 1,000 bootstrap replicates in MEGA 6.0. The jpHMM and RIP 3.0 online tools were used to analyze recombination breakpoints of the two NFLGs.

## Results

**Fig. 1:**
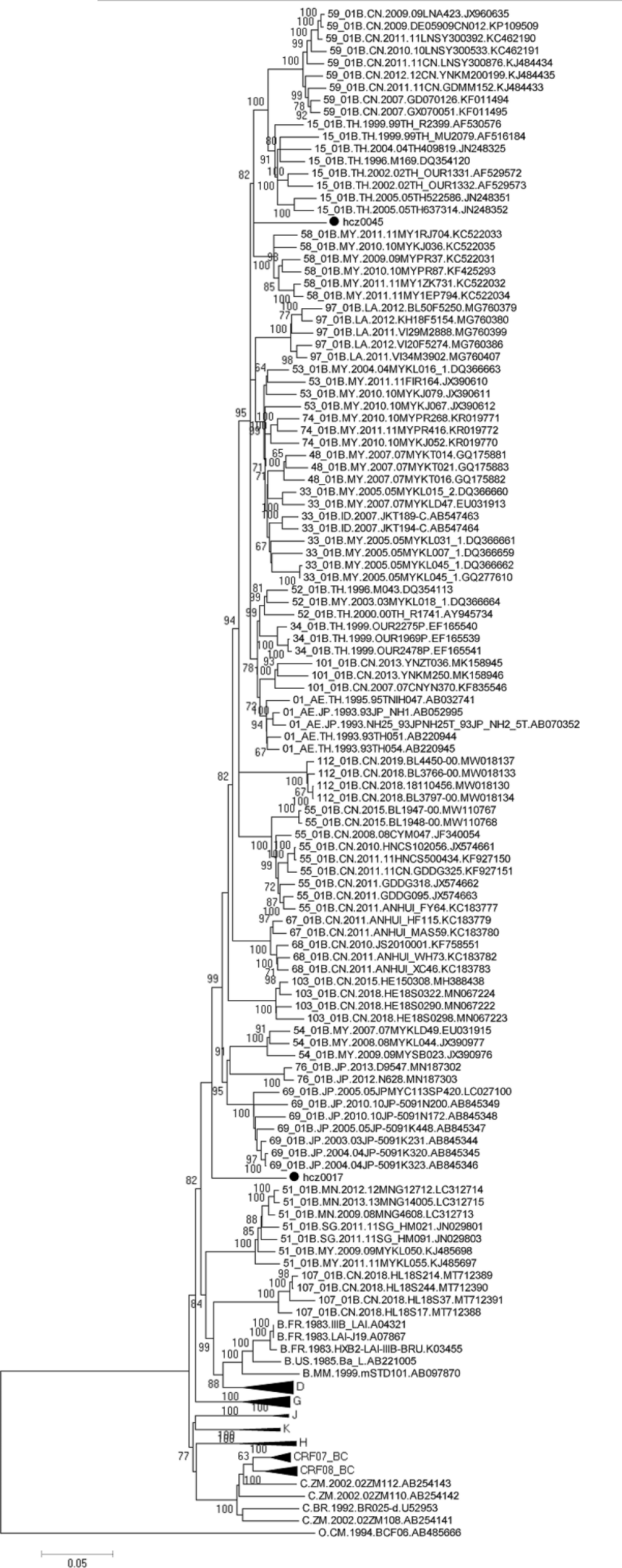
Phylogenetic tree analysis of HIV-1 NFLG sequences. A neighbor-joining tree was constructed using MEGA 6.0, with 1,000 bootstrap replicates. The standard HIV-1 subtype reference sequences were downloaded from HIV Databases (http://www.hiv.lanl.gov/content/index). Bootstrap values ≥ 60% are shown in the tree. The scale bar indicates 5% nucleotide sequence divergence. Each black dot denotes a study subject

**Fig. 2:**
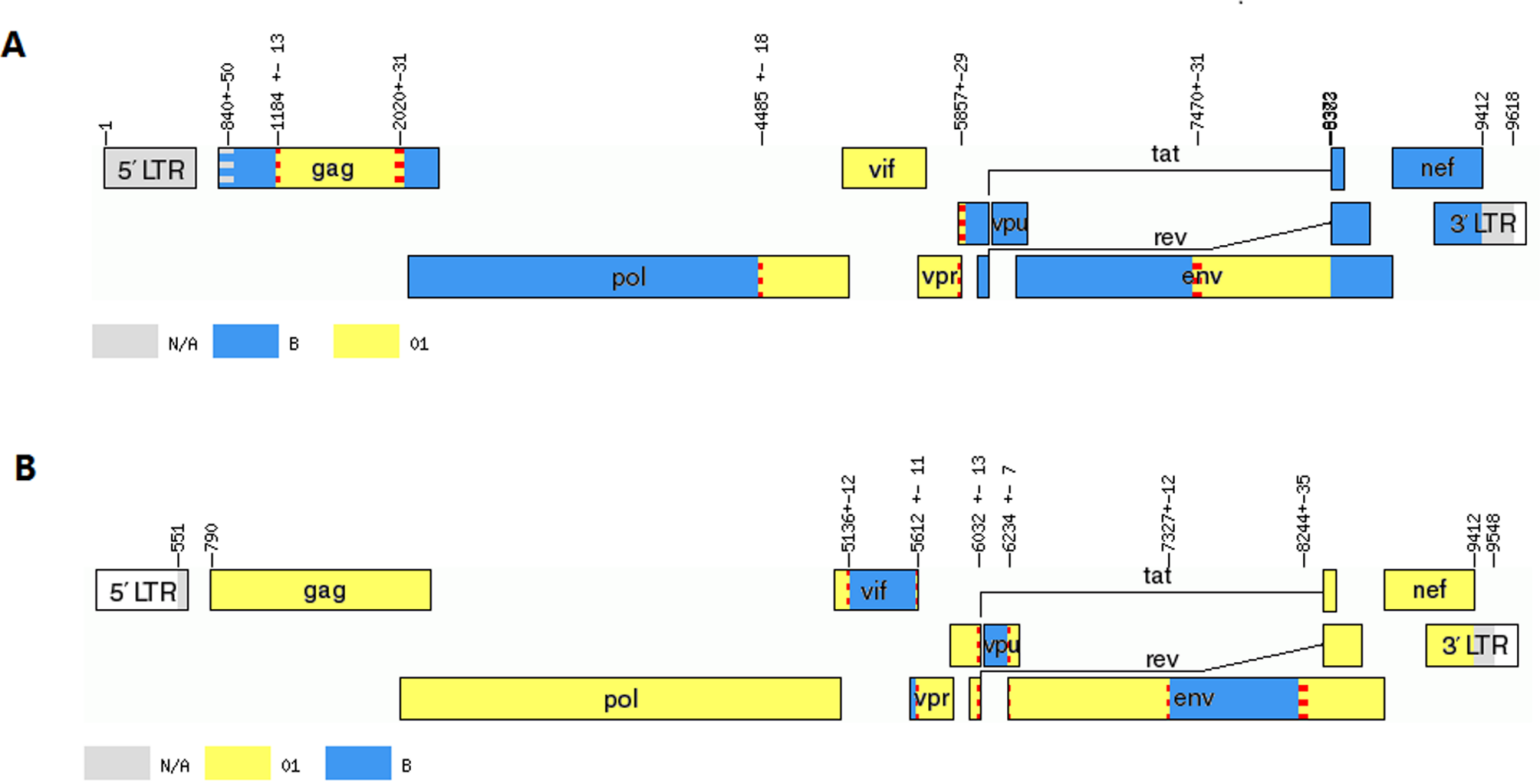
Recombination breakpoint analyses of hcz0017 **(A)** and hcz0045 **(B)**. The NFLG mosaic maps were generated using the online tool jpHMM (http://jphmm.gobics.de/)

**Fig. 3:**
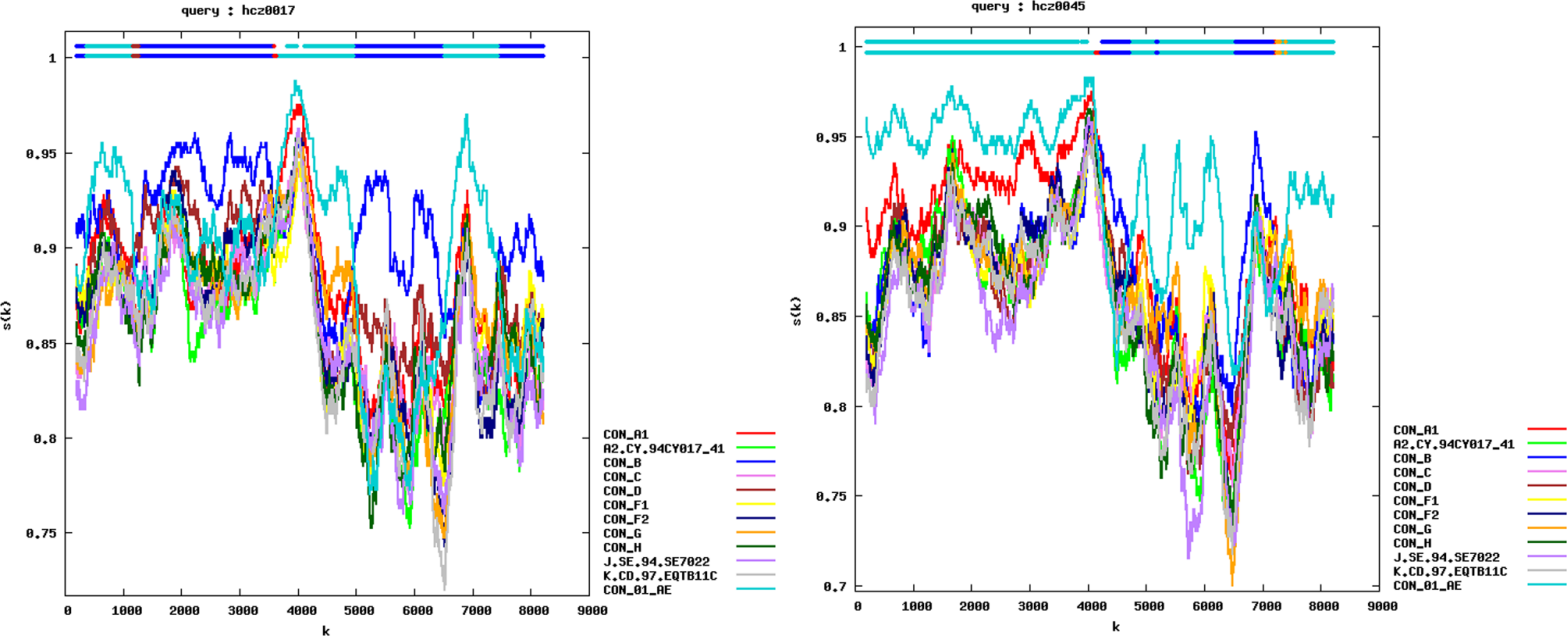
RIP analysis of the hcz0017 and hcz0045 NFLG sequences. Similarity distance analysis was performed using RIP (version 3.0; Siepel AC, Halpern AL, Macken C, Korber BT, http://hiv-web.lanl.gov) from the Los Alamos National Laboratory HIV Database with default settings, except for a window size of 300. Color images are available in the online version of this manuscript

We found that hcz0017 and hcz0045 formed a distinct monophyletic branch, separately from other subtypes and CRFs in the NFLG N-J phylogenetic tree (Fig. 1), suggesting that these two NFLG sequences were novel recombinant forms. According to the results of the jpHMM and RIP 3.0 breakpoint analysis, the hcz0017 and hcz0045 NFLG sequences originated from a recombination between CRF01_AE and subtype B (Figs. 2 and 3) and each contained seven subregions. The hcz0017 NFLG was composed of three CRF01_AE fragments inserted to a subtype B backbone, while the hcz0045 NFLG was composed of three subtype B fragments within a CRF01_AE backbone.

**Fig. 4:**
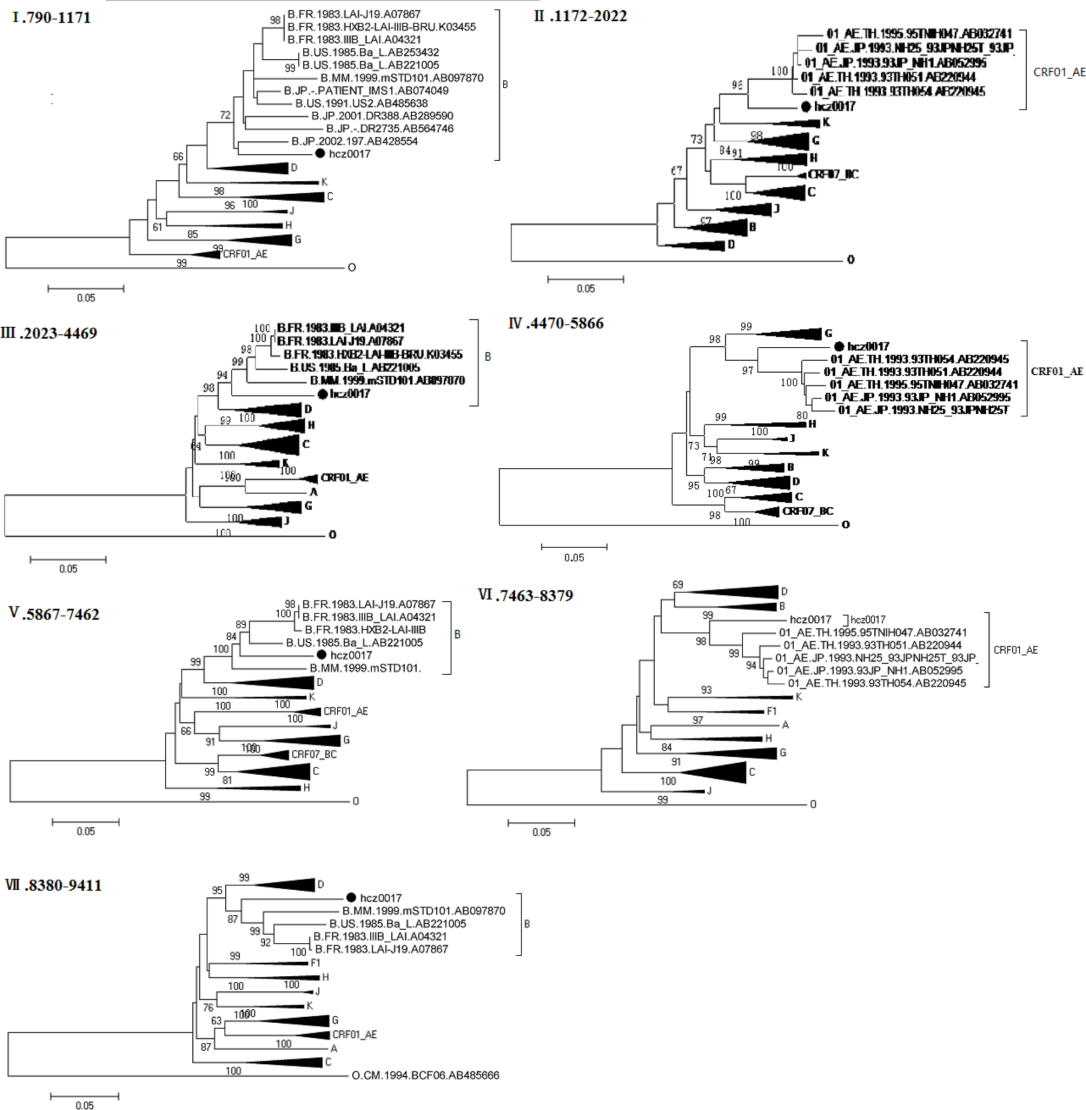
Subregion phylogenetic tree of hcz0017. A neighbor-joining tree was constructed using MEGA 6.0, with 1,000 bootstrap replicates. Each black dot denotes a study subject. Bootstrap values ≥ 70% are shown at the corresponding nodes. The scale bar indicates 5% genetic distance

**Fig. 5:**
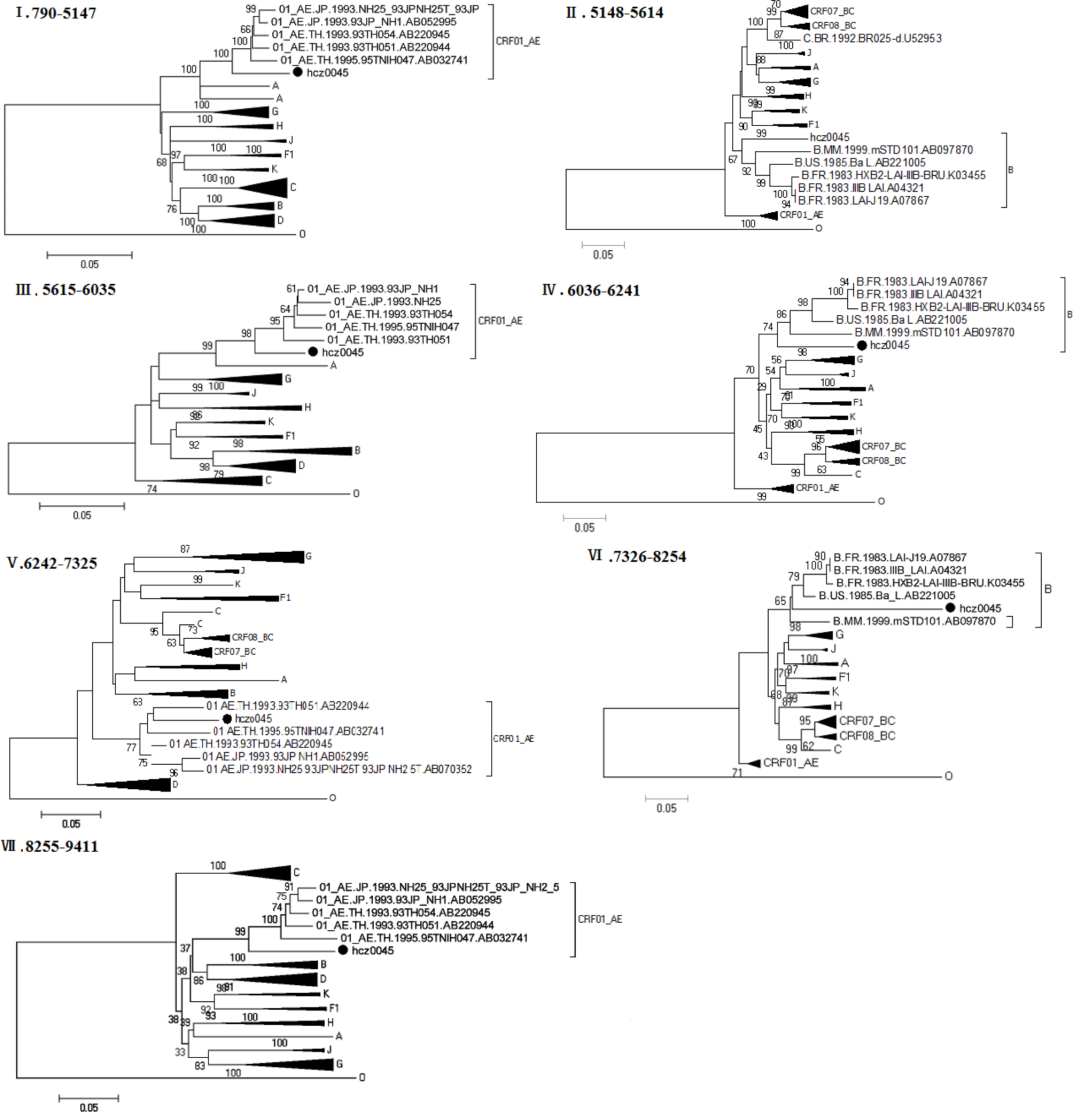
Subregion phylogenetic tree of hcz0045. A neighbor-joining tree was constructed using MEGA 6.0, with 1,000 bootstrap replicates. Each black dot denotes a study subject. Bootstrap values ≥ 70% are shown at the corresponding nodes. The scale bar indicates 5% genetic distance

Next, we performed a detailed analysis of the hcz0017 and hcz0045 NFLG sequence mosaic structures (Figs. 2 and 3). The following positions were assigned to the seven subregions of each URF according to the HXB2 numbering system: (1) hcz0017: I_B_ (790–1,171 nt), II_CRF01_AE_ (1,172–2,022 nt), III_B_ (2,023–4,469 nt), IV_CRF01_AE_ (4,470–5,866 nt), V_B_ (5,867–7,462 nt), VI_CRF01_AE_ (7,463–8,379 nt), VII_B_ (8,380–9,411 nt); (2) hcz0045: I_CRF01_AE_ (790–5,147 nt), II_B_ (5,148–5,614 nt), III_CRF01_AE_ (5,615–6,035 nt), IV_B_ (6,036–6,241 nt); V_CRF01_AE_ (6,242–7,325nt), VI_B_ (7,326–8,254 nt), VII_CRF01_AE_ (8,255–9,411 nt). The phylogenetic tree analysis (Figs. 4 and 5) based on each subregion of each of the NFLGs revealed that all CRF01_AE and B subregions in hcz0017 and hcz0045 clustered with their respective reference sequences. This also revealed the NFLG mosaic structures of the two novel, second-generation recombinant HIV-1 forms. Furthermore, all CRF01_AE and subtype B fragments within the two URFs were closely associated with the Thai-CRF01_AE and Euro-American subtype B, respectively, which have been prevalent in Hebei for 30 years.

## Discussion

18.6% (22/118) of the CRFs listed in the HIV databases have originated from the recombination between CRF01_AE and subtype B. Moreover, six of these were found in MSM, including CRF55_01B, CRF67_01B, CRF69_01B, CRF59_01B, CRF68_01B, and CRF103_01B. Of the above six CRFs, five were found in China and one (CRF69_01B) in Japan. Indeed, novel, second-generation recombinant forms of HIV-1 have been identified continually in some prefectures of Hebei such as Baoding [12], Handan [13], and Shijiazhuang [14]. In this study, the identification of two CRF01_AE/B recombinant forms, with intricate genomic mosaic structures, highlights the complexity of HIV-1 genetics in Cangzhou Prefecture. Moreover, the two MSM in whom the CRF01_AE/B recombinant forms were diagnosed with recent HIV-1 infection, implying a high level of promiscuity among MSM in this region. Our previous study [15] showed that the transmission of HIV-1 occurred among MSM from neighboring provinces, including Beijing, Tianjin, and Hebei. At present, homosexual contact is the most frequent transmission route in the Beijing-Tianjin-Hebei region. Thus, HIV-1 diversity should be constantly monitored in Hebei and its neighboring provinces to more effectively control the spread of HIV-1 among MSM.

## Conclusions

In our study, two novel URFs, which originated from a recombination between CRF01_AE and subtype B, were identified in two recently infected MSM based in Cangzhou Prefecture, Hebei, China by NFLG sequence analysis. Our results highlight the need to continually monitor HIV-1 diversity in Hebei and its neighboring provinces to achieve a more effective control of HIV-1 spread within the MSM community.

## Data Availability

The sequences reported in this study have been submitted to GenBank under accession numbers OK392124 and OK392125.

## Abbreviations

HIV-1: Human immunodeficiency virus type 1
MSM: Men who have sex with men
URF: Unique recombinant form
CRF: Circulating recombinant form
AIDS: Acquired immune deficiency syndrome
NFLG: Near full-length genome

## References

[1] Zheng YY (1988). First case of AIDS diagnosed in China. NatI Med J Chin.

[2] UNAIDS. 2021 Progress Report on the Global AIDS response. Available from: https://www.sohu.com/a/477822682_121106902.

[3] HIV sequence database. : HIV Circulating Recombinant Forms (CRFs). Available at sequence/HIV/CRFs/CRFs.html, accessed September 10, 2018.

[4] Joris H, Ramyiadarsini E, Jason Y, Leslie D, Isabella F, Shona K (2019). Global and regional molecular epidemiology of HIV-1, 1990–2015: a systematic review, global survey, and trend analysis. Lancet Infect Dis.

[5] He X, Xing H, Ruan Y, Hong K, Cheng C, Hu Y (2012). A comprehensive mapping of HIV-1 genotypes in various risk groups and regions across China based on a nationwide molecular epidemiologic survey. PLoS ONE.

[6] Taylor BS, Sobieszczyk ME, McCutchan FE, Scott MH (2008). The challenge of HIV-1 subtype diversity. N Engl J Med.

[7] Teng X, Wang. A summary of HIV/AIDS epidemic situation across the country in 2020. Available from: https://xw.qq.com/amphtml/20201207A0B1EE00. 2020-12-07.

[8] Lu X, Zhao C, Wang W, Nie C, Zhang Y, Zhao H (2016). HIV-1 genetic diversity and its distribution characteristics among newly diagnosed HIV-1 individuals in Hebei province, China. AIDS Res Ther.

[9] Lu X, Kang X, Liu Y, Cui Z, Guo W, Zhao C (2017). HIV-1 molecular epidemiology among newly diagnosed HIV-1 individuals in Hebei, a low HIV prevalence province in China. PLoS ONE.

[10] Wang, Yi. HIV/AIDS epidemic situation across Cangzhou Prefecture in 2020. Available from: http://hebei.news.163.com/20/1127/15/FSEU6A2R0415987E.html. 2020-11-27.

[11] Zhang F, Liang H, Zhong S, Qin C, Yang Y, Liang N (2021). Identification of a HIV-1 CRF01_AE/CRF07_BC unique recombinant form in Guangxi. Chin J AIDS STD.

[12] Zhou J, Lu X, Feng Y, Li M, Zhu Y, Kang R (2020). Genome sequence of a Novel HIV-1 circulating recombinant form (CRF103_01B) identified from Hebei Province, China. AIDS Res Hum Retroviruses.

[13] Xing Y, Guo Y, Wang L, Li H, Wang X, Liu Y (2021). Identification of two Novel HIV-1 Second-Generation recombinant forms (CRF01_AE/CRF07_BC) in Hebei, China. AIDS Res Hum Retroviruses.

[14] Han L, Li H, Wang L, Jia L, Han J, Li T (2021). Near full-length genomic characterization of two Novel HIV-1 unique recombinant forms (CRF01_AE/CRF07_BC) among MSM in Shijiazhuang City, Hebei Province, China. AIDS Res Hum Retroviruses.

[15] Lu X, Zhang J, Wang Y, Liu M, Li Y, An N (2020). Large transmission clusters of HIV-1 main genotypes among HIV-1 individuals before antiretroviral therapy in the Hebei Province, China. AIDS Res Hum Retroviruses.

